# pubCounteR: an R package for interrogating published literature for experimentally-derived gene lists within a user-defined biological context

**DOI:** 10.3389/fbinf.2025.1523184

**Published:** 2025-05-06

**Authors:** Marina Leer, George A. Soultoukis, Markus Jähnert, Masoome Oveisi, Dirk Walther, Tim J. Schulz

**Affiliations:** ^1^ Department of Adipocyte Development, German Institute of Human Nutrition Potsdam-Rehbrücke (DIfE), Nuthetal, Germany; ^2^ German Center for Diabetes Research (DZD), Neuherberg, Germany; ^3^ Department of Experimental Diabetology, German Institute of Human Nutrition Potsdam-Rehbrücke (DIfE), Nuthetal, Germany; ^4^ Bioinformatics, Max Planck Institute of Molecular Plant Physiology, Potsdam, Germany; ^5^ Institute of Nutritional Science, University of Potsdam, Potsdam, Germany

**Keywords:** text mining, literature search, publication activity, gene list, information retrieval

## Abstract

Basic and clinical biomedical research relies heavily on modern large-scale datasets that include genomics, transcriptomics, epigenomics, metabolomics, and proteomics, among other “Omics”. These research tools very often generate lists of candidate genes that are hypothesized or shown to be responsible for the biological effect in question. To aid the biological interpretation of experimentally-obtained gene lists, we developed pubCounteR, an R-package and web-based interface that screens publications by a user-defined set of keywords representing a specific biological context for experimentally-derived gene lists.

## Introduction

Presently, there is a lack of medium-throughput literature survey methods or software to assess publication activity simultaneously for experimentally-derived gene sets in tailored biological contexts defined *a priori*. As modern Omics approaches generate an increasing wealth of available data that entail gene lists in various contexts (e.g., differentially expressed or co-expressed genes, enriched gene sets in regulatory networks, ontologies, or pathways), novel user-friendly tools are needed to enable the rapid assessment of gene lists and published information content.

To facilitate literature searches based on the simultaneous entry of multiple genes in combination with fully customizable search terms for a defined scientific area of interest, we developed pubCounteR, which performs a PubMed database search and returns an annotated and quantified overview of the publication activity for each query gene in a customized user-defined context. pubCounteR can assess medium-sized gene sets through generation of a systematic and biological context-embedded literature overview, to help reveal underlying scientific implications by linking gene-specific published evidence with novel or unfamiliar areas of research. Our package therefore aids the generation of new hypotheses, as well as the interpretation and understanding of experimentally-derived gene lists.

The National Center for Biotechnology Information (NCBI) offers a website (https://www.ncbi.nlm.nih.gov/) featuring search modes that use an Advanced Search Builder interface (Fatehi et al., 2014). Current commonly used search tools used in literature analysis for individual genes employ approaches that predominantly utilize NCBI’s PubMed database to allow users to analyze publication trends per publication year (Serrano et al., 2021), total number of publications per gene (von Mering et al., 2005), or per gene frequency in published gene sets for functional predictions (Clarke et al., 2024), among others. In addition, many disease terms, such as, for example, “diabetes”, “glycogen storage disease”, or “obesity”, are not included in the most common and comprehensive source used for the biological interpretation of gene sets, the Gene Ontology (GO) system (Ashburner CAB et al., 2000; Gene Ontology et al., 2023). To address this gap, disease names obtained from relevant databases such as the Medical Subject Headings (MeSH) can be employed to predict per gene-disease associations (Narganes-Carlon et al., 2023). However, gene-related literature surveys that utilize terms beyond disease states, gene ontologies, or pathways, such as, for example, relating to terms based on analytical methodologies (e.g., “epigenomics”, “single cell”, “GWAS”, etc.), and the parallel query of multiple genes, remain impractical and time-consuming. Thus, currently, such searches are typically performed manually, and there are not presently any tools available to facilitate such approaches.

In contrast to other existing methods, pubCounteR combines a medium-throughput gene input platform and a flexible range of user-defined input search terms, including keywords representing customized biological terms that are currently annotated poorly in existing gene-analytical tools. As such terms (see examples above) are often included in original research articles focusing on specific genes, pubCounteR offers flexibility in linking keyword terms that are not entered in gene-analytical databases with associated research-based literature. This can aid researchers in making direct associations and revealing new scientific implications in diverse areas, including fields where a researcher’s own expertise may be limited. Conversely, in cases where a pubCounteR query returns no current citations linking a particular gene to a user-defined search term, this lack of association could indicate a gap in current research knowledge, whereby this association has either not been studied, or not been published. Therefore, pubCounteR also provides a platform for the identification of gaps in research areas for specific genes. This could help to identify novel genes of interest due to an absence of association with published literature for the keyword-defined biological content. In summary, the principal value of this tool is in supporting a high-throughput biological interpretation of experimentally-obtained gene sets through a systematic and context-embedded literature overview.

## Methods

**FIGURE 1:**
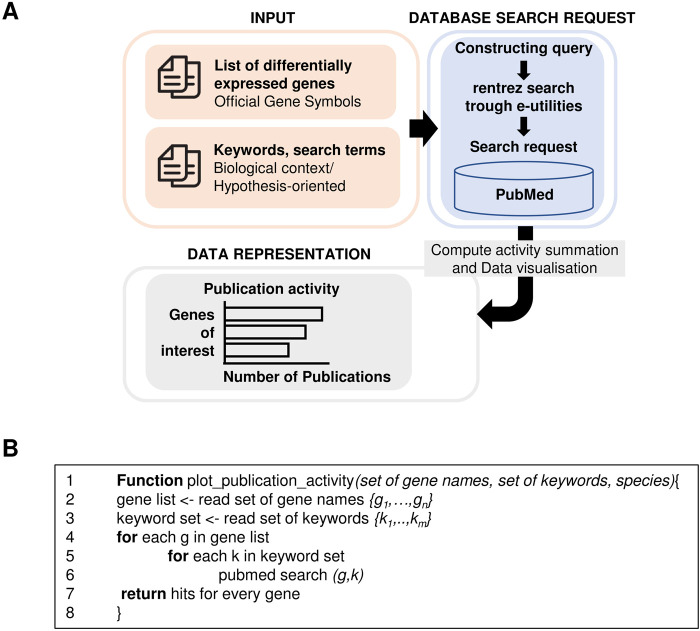
Schematic overview of pubCounteR’s processing workflow and output validation. **(A)** pubCounteR runs a rentrez-query based on all sets of keywords/search terms and each gene entered in a gene list. The R-package then uses the access to NCBI’s PubMed database via a Representational State Transfer Application Programming Interface (REST API). Computing the summation over the publication activity, pubCounteR visualizes for every gene of interest the number of detected publications, ranked by publication numbers in a bar plot. **(B)** Pseudocode for the pubCounteR algorithm. Given a set of genes {g1, … ,gn} and a defined set of keywords {k1, … ,km}, a query is constructed consisting of the keyword terms for each gene, to run a PubMed database search. The returned hits are summed up for each gene separately and represented as publication activity.

**FIGURE 2:**
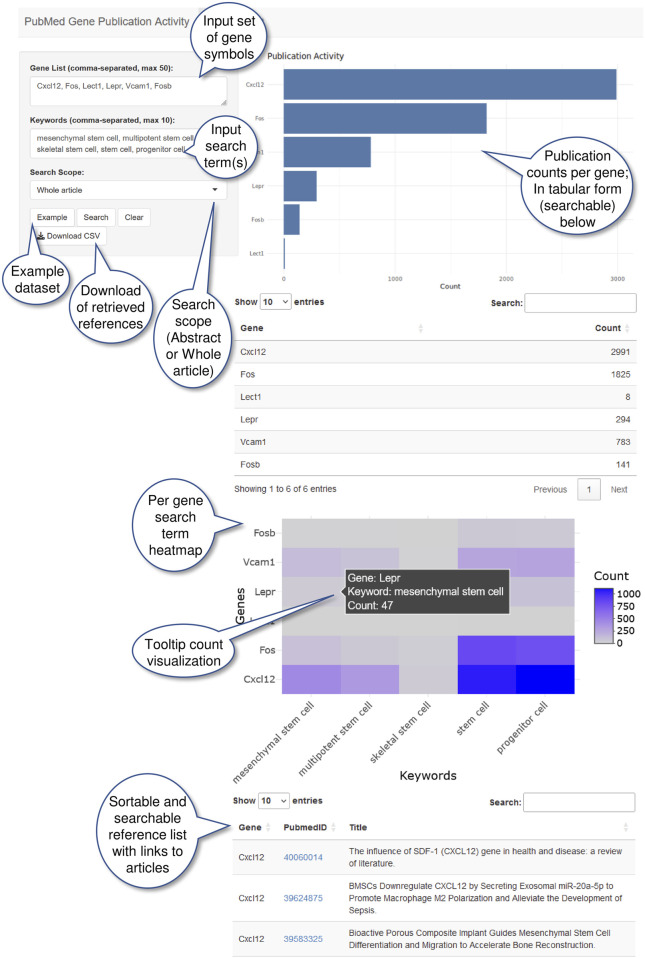
pubCounteR web-based interface (available at https://pubcounter.mpimp-golm.mpg.de/). Annotated screenshot of a pubCounteR query including an example dataset and the required input fields (left panel). Users can insert ≤50 gene symbols of interest, in a comma-separated format, ≤10 keywords as search terms, and define the query scope (abstract or whole article). The generated results pane (right panel) produces a bar chart and table with counts of associated publications per gene, a heatmap visualization per keyword (search term) showing counts per gene, and the most recent 100 research articles related to the gene-keyword associations.

The main workflow of pubCounteR uses as input (i) a list of up to 50 genes of interest, for example, referring to genes found differentially expressed (DEGs) in a particular experiment in official gene symbol format (Maltais et al., 2002), which in the current implementation is confined to human (*Homo sapiens*) and murine (*Mus musculus*) genes, and (ii) an optional set of up to 10 *a priori* defined keyword search terms representing a biological context arranged in a hypothesis generation or biological interpretation style to delineate and/or narrow down the context of gene of interest-specific publication activity. The software then utilizes R (version 4.0.2) and the R-package rentrez (Winter 2017) to conduct a rentrez-search using Entrez Programming Utilities (E-utilities) (Sayers et al., 2023). The R-package then uses the access to NCBI’s PubMed database via a Representational State Transfer Application Programming Interface (REST API). This enables access to Entrez Gene (Maglott et al., 2011), NCBI’s database for gene-specific information, a collection of indexed information on genes from curation and automated analysis by NCBI’s Reference Sequence (RefSeq) project (O’Leary et al., 2016). RefSeq provides access to gene-specific information from the indexed title, abstract, as well as main and supplementary texts of each publication (including indexed table content and citation texts). pubCounteR employs the PubMed database to connect the record of each gene to its corresponding, distinct publications (those assigned unique PubMed identifiers, PMIDs). pubCounteR runs a rentrez-query based on (i) all keyword search terms and (ii) each individual gene separately provided in the gene list of interest, running different iterations for each combination of these two components (Figures 1A,B). As output, it summarizes the number of publications for each gene in an overview bar plot, ranking genes from highest to lowest publication activity (Figure 1A), secondly provides a heatmap of all possible gene - search-term combination, and thirdly generates a summary table in a comma separated value (CSV) format that includes the most recent 100 PMIDs and publication titles for each published article. In an effort to limit individual search runtime, we limit the maximum candidate gene input to 50. Due to the open access structure of this software tool, users’ own preferences and needs can be applied to adapt pubCounteR to different search strategies. pubCounteR is made available both as an R-package and web-based interface implemented as a Shiny app. The implementation as a web-based service provides easy-access (menu-driven query-term submission) and expanded functionality by linking out to the respective PubMed entries of the retrieved literature citations. The functionality and layout of the online pubCounteR interface, as outlined above, is shown in Figure 2.

## Results

### Manual validation of biological context computation

**FIGURE 3:**
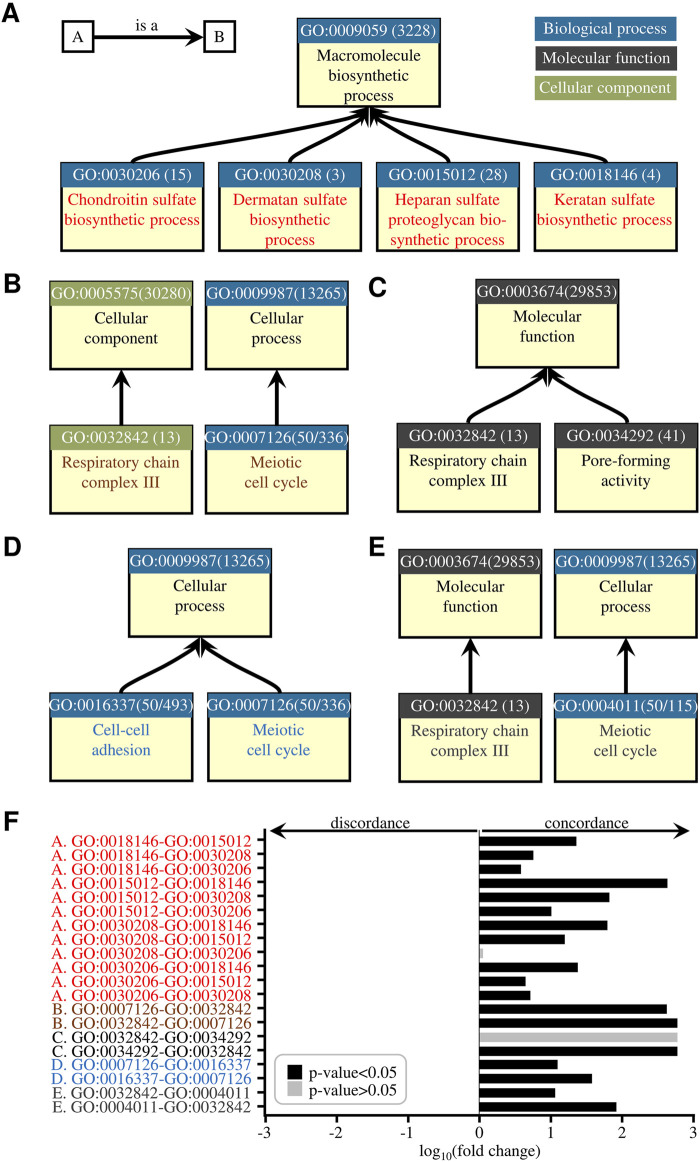
pubCounteR reliably captures and summarizes publication activity for gene sets that reflect existing biological knowledge. **(A)** Node connectivity of four selected gene sets representing a typical hierarchy of relations in the GO system. The GO identifier, number of genes in the term (shown in brackets), and term title are shown. **(B)** Node connectivity of two nodes representing an inter-domain comparison example of a typical hierarchy of relations in the GO system. **(C)** and **(D)** Node connectivities representing intra-domain comparisons. **(E)** As in **(B)**. **(F)** Quantification of the pubCounteR-computed publication activity summation across the GO-organized gene lists shown in Figures 3A–E. Bars represent fold change (in logarithmic scale) of pubCounteR-computed articles as the mean value of all respective genes in each list. Letter code **(A–E)** and font colour indicate the compared child terms in the respective node representations (Figures 3A–E). In the node representations (Figures 3A–E) black font indicates the parent terms, which are shown for clarity of hierarchy and domain structures. Concordance is defined as a change in the mean value that positively reflects the GO term association, and discordance as a change in the mean value that is opposite to the GO term association. p-values represent Mann-Whitney-U test with Bonferroni correction.

To demonstrate the utility of pubCounteR in identifying published literature in connection to defined gene-related biological search terms, we first used as a reference the biological content information organized by the Gene Ontology (GO) system as an example (Ashburner CAB et al., 2000; Gene Ontology et al., 2023). The GO currently includes gene-related biological information based on experimental findings from >150,000 peer-reviewed studies, corresponding to ∼700,000 experimentally-supported annotations and >6,000,000 inferred functional annotations across >5,000 species (Ashburner CAB et al., 2000; Gene Ontology et al., 2023). Using mouse (*M. musculus*) as an example species, which currently contains >160,000 experimentally-supported annotations (Ashburner CAB et al., 2000; Gene Ontology et al., 2023), we first tested four separate sets of genes, each set representing one of four discrete GO terms (GO:0030206 “Chondroitin sulfate biosynthetic process”, GO:0030208 “Dermatan sulfate biosynthetic process”, GO:0015012 “Heparan sulfate proteoglycan biosynthetic process”, GO:0018146 “Keratan sulfate proteoglycan biosynthetic process”) (Figure 3A). These selected four terms share a parent node (GO:0009059 “Macromolecule biosynthetic process”), indicating that although the biosynthetic processes are distinct, they belong to the same macromolecule biosynthetic class and thus are functionally related regarding their underlying biology (Figure 3A). This discrete but also shared node connectivity of the selected four gene sets represents a typical hierarchy of relations in the GO system (Ashburner CAB et al., 2000; Gene Ontology et al., 2023; Binns et al., 2009), making this a suitable exemplary test for validating the accuracy of the functionality of pubCounteR through GO-organized, gene-related publications. For interrogation of existing literature based on selected keyword search terms, we entered input search terms that reflected the defined biological annotation of the gene lists according to the available GO annotation, thereby matching the biological content of GO-organized genes to that of the search terms. Examination of the pubCounteR-compiled publication activity demonstrated both the accuracy and specificity of pubCounteR, as the input search terms robustly reflected the biological content information organized in the GO annotation system (Figure 3F). Moreover, statistically significant results were observed independent of the length of gene lists, from GO terms represented by longer lists (GO:0015012 “Heparan sulfate proteoglycan biosynthetic process”, represented by 28 genes), to short gene lists represented by as few as three (GO:0030208 “Dermatan sulfate biosynthetic process”) or four (GO:0018146 “Keratan sulfate proteoglycan biosynthetic process”) individual genes (Figure 3F; Supplementary Figure S2). The accurate association of search terms to biological content was confirmed across both intra- (Figures 3A,C,D) and inter- (Figures 3B,E) domain comparisons, in the “Biological process”, “Molecular function” and “Cell component” GO domains (Ashburner CAB et al., 2000; Gene Ontology et al., 2023), including comparisons utilizing the currently maximum number of input genes in pubCounteR (fifty), from two “Cellular process” (GO:0050875) terms, namely, “Cell-cell adhesion” (GO:0016337) and “Meiotic cell cycle” (GO:0007126) (Figure 3D). Thus, in our examples, pubCounteR was able to accurately compute the gene-specific biological content for entire input gene lists and reliably matches this content to the biological context provided as an input search term, thereby computing and summarizing with high precision the publication activity for gene sets to reflect existing biological knowledge.

### Confirmation of quantitative precision in research activity computation

The utility of pubCounteR in capturing the research activity for input gene lists was further verified using as an example a gene expression dataset from our previously published study (Ambrosi et al., 2017). The data were obtained in experiments comparing the transcriptomic signatures of defined cell populations occurring in bone tissue as measured by RNA-sequencing. For reference, a subset of bone-resident multipotent mesenchymal stromal cells (MSC) was compared to its cellular progeny, as it can produce daughter cells either committed to undergo differentiation into bone cells (osteogenic progenitor cells; OPC) or fat cells (adipogenic progenitor cells; APC). DEGs enriched in one of the three cell types were determined by statistical significance (p-value <0.05). These cell population-defining marker genes were separated by manual literature inspection, performed by experienced researchers in the relevant field, into two categories, corresponding to (i) established marker genes that are well-documented in the published literature on stem cells, and (ii) novel marker genes which show little, if any, publication activity related to stem cells (Ambrosi et al., 2017) (Supplementary Table S1).

**FIGURE 4:**
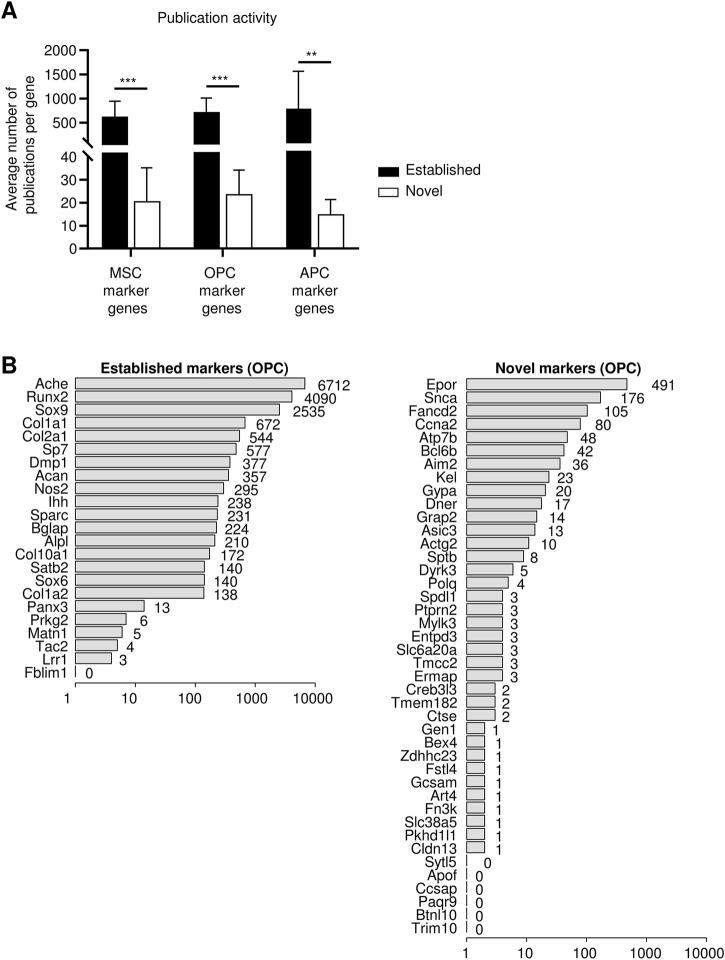
pubCounteR effectively recapitulates the manual assessment of research activities for individual candidate gene entries. **(A)** Average number of publications per gene from a validation of publication activity by pubCounteR. Differentially expressed marker genes of three defined stem and progenitor cell populations as previously identified were used (Ambrosi et al., 2017). A 10-keyword set referring to the research area “Stem Cell Biology” was generated and two lists containing either established or novel marker genes of multipotent mesenchymal stromal cells (MSC), osteogenic (OPC), or adipogenic progenitor cells (APC) were entered (individual genes and keyword set: Supplementary Tables S1, S2). Bars represent average number of pubCounteR-computed articles as mean ± SEM summarizing all respective genes in each list, Mann-Whitney-U test with Bonferroni correction; * <0.01 ** <0.001 *** <0.0001. **(B)** Output plots visualize publication activity in number of publications in logarithmic scale and shown separately for established markers (23 genes; left panel) and novel markers (42 genes; right panel) of OPCs as published in (Ambrosi et al., 2017). This illustrative pubCounteR output plot for the two gene lists (novel and established) from OPCs is accompanied by output examples of the most recent 100 publications for the two OPC gene lists in CSV format in Supplementary Files S1, 2 respectively.

To validate the publication activity through pubCounteR, these lists of marker genes were used as input data, and the number of identified articles per gene was summarized to compare established gene- and novel gene-related publication activities in an unbiased manner. A biologically defined keyword set was used as co-input, referring to the original area of research from which the dataset was derived, i.e., “stem cell biology” (Supplementary Table S2). pubCounteR analysis showed that genes previously annotated as established marker genes in the reference dataset (Ambrosi et al., 2017) displayed higher average publication activity when compared to the novel marker gene category, confirming the ability of our tool to identify established genes through a higher published literature content (Figure 3A). By contrast, genes previously annotated manually as novel markers consistently showed a significantly lower average publication activity for all three cell types (Figures 4A,B).

Lastly, the runtime of pubCounteR was found to scale linearly with input size (number of entry genes; Supplementary Figure S1A), and search duration was increased when genes with high publication activity were entered (Figure 1B). To conclude, our examples demonstrate that pubCounteR detects with quantitative precision the published gene-specific context-based research activity, thereby providing a dependable depiction of the existing volume of publication activity for input gene sets in relation to the user-defined biological context.

## Discussion

There are currently no literature survey methods or software for entire experimentally-derived gene sets to assess publication activity in specific biological contexts user-defined *a priori*. NCBI offers an advanced user interface featuring multiple search modes and filters allowing literature interrogation by date, author, gene name, and user-defined search term, among others (Fatehi et al., 2014). However, for multiple genes, in which a query on a gene list and one or multiple user-defined search terms are used as input, the interface only returns cumulative results. In consequence, the parallel assessment of the existing literature for multiple individual candidate genes requires manual input and therefore remains time-consuming when a list of multiple genes is to be evaluated, in particular with regards to user-defined biological contexts and quantitative per gene-outputs.

We here present a novel literature assessment software that quantitatively summarises published research for multiple individual candidate genes filtered by a user-defined and keyword-based specific biological area of interest. In contrast to these other existing methods, pubCounteR combines a medium-throughput gene input and a broad and fully flexible range of user-defined input search terms, including keywords representing other customised biological terms that are currently annotated poorly in existing gene-analytical tools or databases. This is highlighted in Figure 4 by the search term “osteochondrogenic progenitor cell”, a term representing a highly specific biological feature that is not found in neither GO or MeSH terms. Given the cumulative and accelerating nature of basic and clinical research data collection, and the rapid advancement of new fields of research, many researchers study cutting edge biological concepts or phenomena that may be equally lacking representation in common databases. However, such terms are often included in original research articles focusing on specific genes in the context of state-of-the-art or specialised niche research. Hence, pubCounteR offers increased flexibility in linking less common terms that are under-represented in current gene-analytical databases with associated research-based literature. This can aid researchers to make direct associations and reveal new scientific implications in diverse and novel areas of research. Thus, due to the open access structure of this software tool, users’ own preferences and needs can be applied to adapt in different search applications.

Other existing tools for the interrogation of biological research literature predominantly utilize the PubMed database in order to quantify the latest publications per year (Serrano et al., 2021), the per gene total number of publications (von Mering et al., 2005), or the frequency of individual genes in published gene sets for predicting putative functions (Clarke et al., 2024). Biological interpretation of gene sets is also derived from databases such as the Gene Ontology knowledgebase (Ashburner CAB et al., 2000; Gene Ontology et al., 2023). Other databases such as the Medical Subject Headings are also utilised by literature-interrogating tools to infer disease associations for gene sets (Narganes-Carlon et al., 2023), or to use co-citation networks in order to associate gene sets to biological pathways and disease-related MeSH terms (Hu et al., 2020). However, the interrogation of gene sets on the basis of user-defined search terms, with the aim of visualising associations of genes to custom keywords, is currently not available because, to our knowledge, none of the existing tools accommodate a function for a user-defined search term as input for making associations to a gene set. This also means that direct comparisons of pubCounteR cannot be made to existing tools, as their functions serve different purposes, and apply different methodologies. As no other current approaches perform this task, pubCounteR fills in a significant gap, thereby enabling a novel method for gene-based scientific literature visualisation.

Large Language Models (LLMs) represent a major new development for text processing (Birhane et al., 2023). However, pubCounteR serves a specific purpose, namely, to perform gene queries in conjunction with search terms that capture specific biological contexts. In this case, an LLM in fact would not be a viable alternative, as a purely quantitative output would have to be strictly defined. Moreover, LLMs depend entirely on their training dataset, which is often environmentally costly and sourced from various potentially unreliable or outdated materials that may contain intrinsic biases (Birhane et al., 2023; Navigli et al., 2023). Consequently, such a model would not necessarily reflect the most accurate or up-to-date information, and would introduce redundancy at the expense of complexity, resulting in missing information and potentially leading to false conclusions (Birhane et al., 2023; Navigli et al., 2023). This is especially important, given that one of the main utilities of pubCounteR is intended to be for use in novel and niche scientific research areas that are not represented currently in common analytical databases (e.g., databases such as GO or MeSH). In contrast to LLMs, pubCounteR connects directly to PubMed, ensuring access to the most current information available today, as scientific data are updated daily. LLMs are also prone to hallucination, generating inaccurate or misleading information (Birhane et al., 2023; Navigli et al., 2023). This is particularly concerning when dealing with scientific data, where precision and reliability are paramount. Moreover, even LLMs that use generative search engines to rely on up-to-date information perform poorly in quantitative reliability tests within biomedical knowledge (Phan et al.,2025; Liu et al., 2023), as they rely on web searches rather than on database interrogations. The mismatch between data actuality, the use of reliable source databases, avoiding any data selection bias in training, and the environmental costs of creating a task-specific LLM for retrieving high-quality scientific data automatically strongly advise using pubCounteR instead of a trained LLM. Thus, the applications of the tool are somewhat related, but in our view also different to what an LLM method could currently provide. pubCounteR can quantify the associations of genes or gene sets to user-defined terms, while also relying on a defined source of information input. Although the functionality of pubCounteR could potentially be incorporated into future AI tools, here, we exploit the specific rentrez search functionality to retrieve citations based on specific search fields and text positions (Full text vs. Abstract) in a fully quantitative manner with defined input and output parameters.

In summary, pubCounteR’s principal value is in supporting biological interpretations of experimentally-obtained gene sets through customisable, context-embedded literature overviews, enabling the visualisation of novel gene-keyword associations, including for search terms that are not (yet) curated in established knowledgebases, or in fields where a researcher’s own expertise may be limited. In addition, it allows for the identification of gene-specific research gaps as it helps establish novel genes of tentative interest through their lack of specific biological content associations in user-defined novel research fields where published literature is absent, for instance in the context of otherwise well-studied candidate genes.

## Data Availability

The original contributions presented in the study are included in the article/Supplementary Material, further inquiries can be directed to the corresponding authors.

## Supplementary material

SUPPLEMENTARY FIGURE S1
Effect of input size parameters on runtime (in seconds) of a pubCounteR analysis. **(A)** Effect of number of genes as an input size parameter, independent of established or novel gene characterisation. **(B)** Effect of the number of genes as an input size parameter, shown separately for established genes and novel genes. Solid lines connect the individual data points, dotted lines represent the respective best-fit lines from linear regression fits, grey error bars represent the 95% confidence intervals, y represents the slope, *R* ^2^ is the coefficient of determination, and the p-value represents a two-tailed test of the null hypothesis that the slopes are identical.

SUPPLEMENTARY FIGURE S2
(related to main Figure 3): Illustration of the pubCounteR quantification of publication activity summation across the GO-organized gene lists. Examples shown for node term association analyses of Figures 3A,D. **(A)** Violin plot visualization for the nodes shown in Figure 3A, and the concordance visualization in Figure 3F, with the keyword search terms applied also shown as key. **(B)** As in **(A)** but for the nodes shown in Figure 3D. A similar approach was applied for the node term association analyses of Figures 3B–E.
