# Supplementary material for: pubCounteR: an R package for interrogating published literature for experimentally-derived gene lists within a user-defined biological context
Source: Front Bioinform. 2025 May 6;5:1523184. doi: 10.3389/fbinf.2025.1523184 (PMC12118352; doi:10.3389/fbinf.2025.1523184)
Supplement: Supplementary file 1 [file Table1.docx]

Supplementary Material

# Supplementary Data

Supplementary File 1. Example of an excel output file for established OPC markers, listing the top ≤ 100 publications (by PMID and title) for each of the interrogated genes.

Supplementary File 2. Example of an excel output file for novel OPC markers, listing the top ≤ 100 publications (by PMID and title) for each of the interrogated genes.

# Supplementary Figures and Tables

**Supplementary Table 1.** Cell type-specific marker gene lists.

**Supplementary Table 2.** Keyword set example for biological context definition.

## Supplementary Tables

|  | **Multipotent mesenchymal stromal cells (MSC)** | **Osteochondrogenic progenitor**  **cells (OPC)** | **Adipogenic progenitor cells (APC)** |
| --- | --- | --- | --- |
| **Known marker genes** | Cxcl12, Fos, Lect1, Lepr, Vcam1, Fosb, Pde4b, Scrg1, Myc, Kitl, Osmr, Il1rn, Serpina3g, Il7, Fgf2, Gpr50, Akr1c19, E2f7, Sntb1, Lhx6, Sstr2 | Sparc, Col1a2, Col1a1, Col2a1, Bglap, Alpl, Acan, Col10a1, Panx3, Sox9, Wisp1, Dmp1, Matn1, Prkg2, Sox6, Runx2, Satb2, Sp7, Tac2, Nos2, Ache, Lrr1, Ihh, Fblim1 | Gsn, Dcn, Cav1, Clic5, Igf1, Ebf1, Cd34, Fgl2, Adam17, Scara5, Dkk2, Rhoj, Cpt1a, Dpp4, Ebf2, Col4a2, Gpr1, Col6a6, Zfp423, Elovl7, Entpd2, Gas2l1, Ednrb, Cdh4, Tmem26, Ppara, Nat8, Dlx4, Adam23, Arntl2 |
| **Novel marker genes** | Nog, Serpina3f, Slc2a5, St8sia1, Adra2b, Gdf6, Fam155a, Tmprss2, Ttpa, Syt14, Erv3, Wdr86, Scnn1b, Lypd6b, Mbl1, Tmem171, Clstn3, Alpk2, Pof1b, Tmem125, Fbp1, Derl3, Klhl41, Hsh2d, Aloxe3, Veph1, Hcar1, Kcnmb2, Ranbp17 | Ctse, Ermap, Tmcc2, Gypa, Cldn13, Slc38a5, Sptb, Trim10, Kel, Entpd3, Btnl10, Snca, Pkhd1l1, Mylk3, Fancd2, Paqr9, Polq, Epor, Ccna2, Slc6a20a, Fn3k, Art4, Ccsap, Grap2, Tmem182, Atp7b, Apof, Dyrk3, Ptprn2, Asic3, Gcsam, Aim2, Creb3l3, Sytl5, Fstl4, Zdhhc23, Bex4, Bcl6b, Gen1, Spdl1, Dner, Actg2 | Fndc1, Tnxb, Adgrd1, Tspan15, Procr, Megf10, Tmod2, Celf2, Ociad2, Il33, Tmem196, Serpinb7, Irak3, Clip4, Fam124a, C7, Sdk1, Grap, Serpinb8, Impg2, Mst1r, Mblac2, Capns2, Slc4a3, Hoxa11os, Krtdap, Il27ra, Cfap45, Fut9, Tmem151a, Rspo1, Cdhr5 |

**Supplementary Table 1.** Cell type-specific marker gene lists. Markers were subdivided to represent multipotent mesenchymal stromal cells (MSCs) and cells either specialized towards an osteogenic lineage (OPC; osteogenic progenitor cells) or towards an adipogenic lineage (APC; adipogenic progenitor cells). Markers were subdivided into known and novel gene categories, based on manual literature evaluation and as published in (13).

| **Keyword Set: Stem Cell Biology** | | | |
| --- | --- | --- | --- |
| chondrogenic  stem cell | osteogenic  stem cell | adipogenic  stem cell | osteogenic progenitor cell |
| adipogenic progenitor cell | mesenchymal stromal cell | mesenchymal  stem cell | multipotent  stem cell |
| skeletal stem cell | stem cell | progenitor cell |  |

Supplementary Table 2. Keyword set example for biological context definition. The keyword set representing example terms used to restrict the analysis to the field of bone tissue-resident stem/stromal cell biology.
