# Supplementary figures and images for: pubCounteR: an R package for interrogating published literature for experimentally-derived gene lists within a user-defined biological context
Source: Front Bioinform. 2025 May 6;5:1523184. doi: 10.3389/fbinf.2025.1523184 (PMC12118352; doi:10.3389/fbinf.2025.1523184)

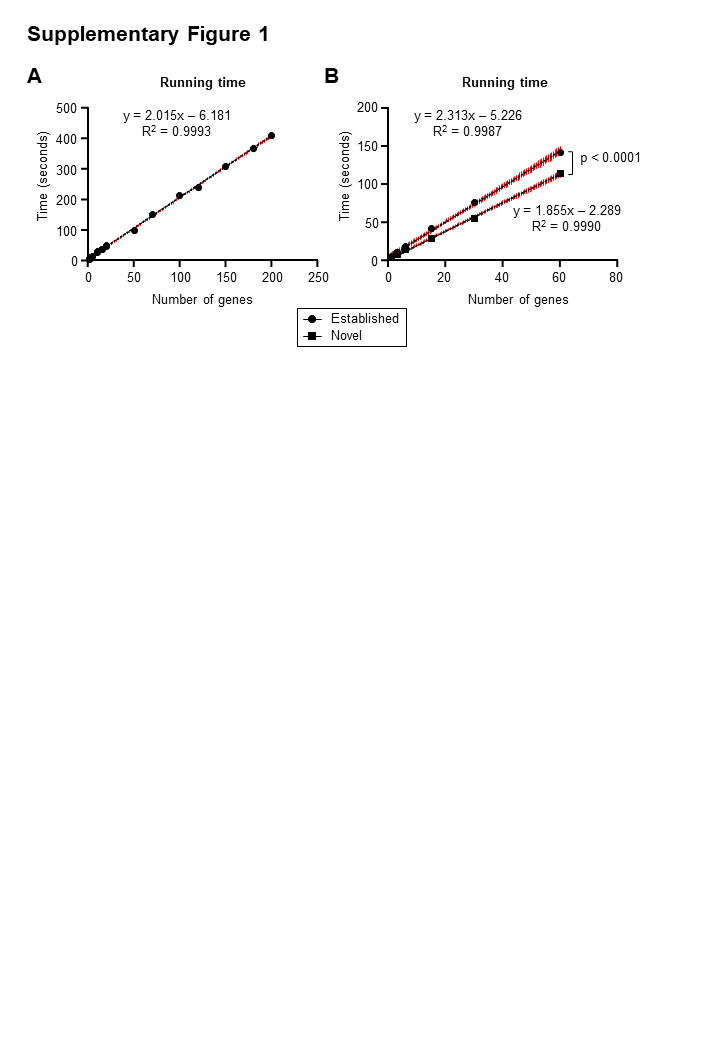

Supplement: Supplementary file 2 [file Image1.tif]
